# Skewing of the TCR V repertoire in SMX-NO specific T cell responses

**DOI:** 10.1186/2045-7022-4-S3-P114

**Published:** 2014-07-18

**Authors:** Lee Faulkner, Maike Lichtenfels, Philipp Esser, Stefan Martin, Ana Alfirevic, Munir Pirmohamed, Dean Naisbitt, Kevin Park

**Affiliations:** 1 University of Liverpool, MRC Centre for Drug Safety Science, UK; 2 University Medical Centre, Allergy Research Group, Germany

## Background

A role for drug-specific T cells has been demonstrated in many instances of drug hypersensitivity. In the case of -lactam antibiotics, such as flucloxacillin, we have shown that drug-protein conjugates are present in the plasma of all patients treated with this antibiotic, however only a small subset of patients develop hypersensitivity. Similarly, when a strong HLA association to drug hypersensitivity has been demonstrated such as for abacavir, not all patients with the risk allele will develop a hypersensitivity reaction. The mechanism underlying this variation in susceptibility is not fully understood. In carbamazepine-induced Stevens Johnson syndrome it is known that only patients with both the HLA risk allele and T cells with a specific TCR V will develop a reaction. This shows that an individual's T cell repertoire may confer some susceptibility to drug hypersensitivity.

## Method

In initial experiments, SMX-NO-specific CD4+ clones were generated from hypersensitive patients and analysed for TCR V expression. Nitroso sulfamethoxazole (SMX-NO)-specific T cells were generated from 6 individuals by priming naïve T-cells using drug-treated autologous dendritic cells. The responding cells were isolated using positive CD45RO magnetic bead selection. Naïve and SMX-NO specific T cells were analysed by serology for 24 TCR Vs and by RT-PCR spectra-typing of the CDR3 region.

## Results

There was a marked expansion of TCR V4 and V9 in 5 donors, of TCR V11, V13.6 and V14 in 4 donors and of TCR V5.2 and V18 in 3 donors. We were able to show clonal expansion of these individual TCR Vs using CDR3 spectratyping analysis.

## Conclusion

This data suggests that there may be a skewing of the T cell repertoire in SMX-NO specific responses.

